# The Five-To-Six-Coordination Transition of Ferric Human Serum Heme-Albumin Is Allosterically-Modulated by Ibuprofen and Warfarin: A Combined XAS and MD Study

**DOI:** 10.1371/journal.pone.0104231

**Published:** 2014-08-25

**Authors:** Carlo Meneghini, Loris Leboffe, Monica Bionducci, Gabriella Fanali, Massimiliano Meli, Giorgio Colombo, Mauro Fasano, Paolo Ascenzi, Settimio Mobilio

**Affiliations:** 1 Department of Sciences, Roma Tre University, Roma, Italy; 2 National Institute of Biostructures and Biosystems, Roma, Italy; 3 Biomedical Research Division, Department of Theoretical and Applied Sciences, and Center of Neuroscience, University of Insubria, Busto Arsizio (VA), Italy; 4 Institute for Molecular Recognition Chemistry, National Research Council, Milano, Italy; 5 Interdepartmental Laboratory of Electron Microscopy, Roma Tre University, Roma, Italy; Aligarh Muslim University, India

## Abstract

Human serum albumin (HSA) is involved physiologically in heme scavenging; in turn, heme-albumin (HSA-heme-Fe) displays globin-like properties. Here, the allosteric effect of ibuprofen and warfarin on the local atomic structure around the ferric heme-Fe (heme-Fe(III)) atom of HSA-heme-Fe (HSA-heme-Fe(III)) has been probed by Fe-K edge X-ray absorption spectroscopy (XAS). The quantitative analysis of the Fe-K edge extended X-ray absorption fine structure (EXAFS) signals and modeling of the near edge (XANES) spectral features demonstrated that warfarin and ibuprofen binding modify the local structure of the heme-Fe(III). Combined XAS data analysis and targeted molecular dynamics (MD) simulations provided atomic resolution insights of protein structural rearrangements required to accommodate the heme-Fe(III) upon ibuprofen and warfarin binding. In the absence of drugs, the heme-Fe(III) atom is penta-coordinated having distorted 4+1 configuration made by the nitrogen atoms of the porphyrin ring and the oxygen phenoxy atom of the Tyr161 residue. MD simulations show that ibuprofen and warfarin association to the secondary fatty acid (FA) binding site 2 (FA2) induces a reorientation of domain I of HSA-heme-Fe(III), this leads to the redirection of the His146 residue providing an additional bond to the heme-Fe(III) atom, providing the 5+1 configuration. The comparison of Fe-K edge XANES spectra calculated using MD structures with those obtained experimentally confirms the reliability of the proposed structural model. As a whole, combining XAS and MD simulations it has been possible to provide a reliable model of the heme-Fe(III) atom coordination state and to understand the complex allosteric transition occurring in HSA-heme-Fe(III) upon ibuprofen and warfarin binding.

## Introduction

Human serum albumin (HSA), the most abundant protein in plasma (*ca.* 7×10^−4^ M), represents the main determinant of plasma oncotic pressure, is the major modulator of fluid distribution within the body compartments, and displays an extraordinary ligand-binding capacity [1], [2]. Indeed, HSA provides a depot and carrier for many endogenous and exogenous compounds, affects pharmacokinetics of many drugs, induces the metabolic modification(s) of some ligands, renders potential toxins harmless, accounts for most of the anti-oxidant capacity of human plasma, and displays (pseudo-)enzymatic properties [1], [2]. HSA is a single non-glycosylated all-α chain protein, constituted by 585 amino acids, containing three homologous domains (labeled I, II, and III). Each domain is made up by two separate subdomains (named A and B) connected by random coils [2]–[5].

**Figure 1:**
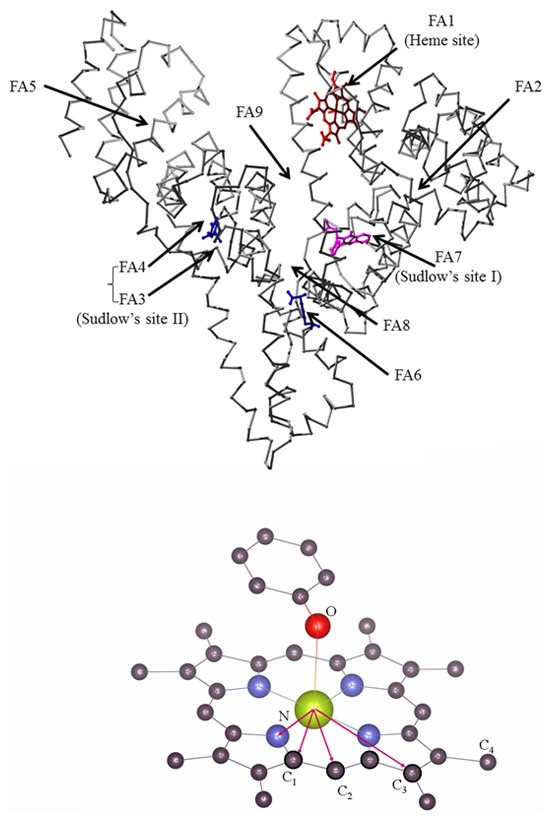
HSA structure. (Top) The heme (red) fits the FA1 site in subdomain IB. Sudlow's site I (in subdomain IIA, corresponding to FA7) is occupied by warfarin (magenta). Sudlow's site II (in subdomain IIIA, corresponding to FA3–FA4) and FA6 (in subdomain IIB) are occupied by ibuprofen (blue). Atomic coordinates were taken from PDB entries 1O9X [22], 2BXD [10], and 2BXG [10], picture drawn using the UCSF Chimera package [67]. (Bottom) The local heme-Fe(III) structure is highlighted, the atoms belonging to the heme-Fe(III) coordination shells are labeled.

The structural organization of HSA provides several ligand binding sites. In particular, HSA displays at least nine fatty acid (FA) binding clefts (FA1–FA9) [6]. The FA1 site (located in subdomains IB) has evolved to specifically bind the heme, the FA3 and FA4 sites compose the so-called Sudlow's site II (located in subdomain IIIA) that recognizes preferentially aromatic carboxylates with an extended conformation, and the FA7 site represents the so-called Sudlow's site I (located in subdomain IIA) that binds especially bulky heterocyclic anions [2], [5], [7], [8], [9]. Remarkably, ibuprofen, a non-steroidal anti-inflammatory drug, and warfarin, a coumarinic anticoagulant drug, are considered to be the stereotypical ligands for the FA3–FA4 cleft and the FA7 site, respectively. Moreover, ibuprofen has been reported to bind also to the FA2 and FA6 secondary sites and warfarin to the FA2 secondary cleft [2], [10]–[14] (Fig. 1).

FA binding to FA2 and FA3 sites, located at the interfaces between subdomains IA and IIA and between subdomains IIB and IIIA, respectively, drives the conformational transition(s) of HSA. In particular, the FA-loaded conformational state of HSA corresponds to the B (*i.e.*, basic) form of the protein, whereas the FA-free HSA is in the N (*i.e.*, neutral) conformational state [2]. It has been demonstrated that the presence of His146 is necessary for the allosteric N-to-B transition [15].

Ibuprofen and warfarin modulate allosterically the heme-albumin (HSA-heme) reactivity and spectroscopic properties [2], [11]–[13], [16]–[18]. Indeed, it has been hypothesized by optical spectroscopy investigations that these drugs may facilitate the formation of the sixth coordination bond of the heme-Fe atom with the His146 residue [11], [13], [14], [19], [20], the fifth coordination ligand of the heme-Fe atom being the Tyr161 residue [21], [22].

In the present study, the local sensitivity and chemical selectivity of X-ray absorption spectroscopy (XAS) technique [23], [24] are exploited to directly probe the modification of local atomic structure around the ferric heme-Fe atom of HSA-heme (HSA-heme-Fe(III)), induced by ibuprofen and warfarin binding. The analysis of Fe-K edge XAS data, consistently with literature data, point out the coordination geometry of the heme-Fe(III) atom in ligand-free HSA-heme-Fe(III) made by the four closely-bound (around 2.0 Å) nitrogen atoms of the protoporphyrin-IX ring and a weakly-bound (around 2.5 Å) oxygen phenoxy atom of Tyr161 residue, giving rise to a distorted 4+1 penta-coordination mode. Upon ibuprofen and warfarin binding, the heme-Fe(III) local coordination changes with an additional neighbor giving rise to a distorted six-coordinated site with the 5+1 configuration. Steered molecular dynamics simulations (SMDS) provide further details on the origin of such additional heme-Fe(III) ligand and, in general, about HSA structural deformations induced by ibuprofen and warfarin. The SMDS models show that ibuprofen and warfarin binding to the FA2 secondary cleft induces a reorientation of domain I leading to the redirection of the His146 residue coordinating the heme-Fe(III) atom at the sixth position. The structure obtained after the SMDS stage was further studied by means of classical molecular dynamics (MD) simulations to probe its viability. In order to prove the validity of the results, the local structure obtained by SMDS has been used as the input for *ab initio* modeling of the heme-Fe(III) atom X-ray absorption near edge structure (XANES) features. The consistency of model and experimental features in the XANES region strengths further the reliability of the proposed structural models.

## Materials

Fatty acid-free HSA, hemin (Fe(III)-protoporphyrin IX) chloride, ibuprofen, and warfarin were purchased from Sigma-Aldrich (St. Louis, MO, USA).

HSA-heme-Fe(III) was prepared by adding a 0.8-molar defect of heme-Fe(III) to the HSA solution (1.0×10^−1^ M sodium phosphate buffer, pH 7.0) at 25.0°C [25]. Under these conditions, no free heme is present in the HSA-heme-Fe(III) solutions; indeed, the dissociation equilibrium constant for heme binding to HSA is 1.3×10^−8^ M and 1.5×10^−7^ M (at pH 7.0 and 25.0°C) in the absence and presence of drugs, respectively [25]. The HSA-heme-Fe(III) concentration was determined spectrophotometrically at 403 nm (ε = 1.1×10^5^ M^−1^ s^−1^) [2]. The final HSA-heme-Fe(III) concentration was 1.0×10^−4^ M.

The ibuprofen stock solution (1.0×10^−1^ M) was prepared by dissolving the drug in 1.0×10^−1^ M phosphate buffer, pH 7.0, at 25.0°C [25]. The final ibuprofen concentration was 1.0×10^−2^ M. The warfarin stock solution (1.0×10^−1^ M) was prepared by stirring the drug in 1.0×10^−1^ M phosphate buffer at pH 12.0 until it dissolved, then adjusting the solution to pH 7.0 with HCl, at 25.0°C [25]. The final warfarin concentration was 1.0×10^−2^ M. In the presence of 1.0×10^−2^ M ibuprofen or warfarin, HSA-heme-Fe(III) was fully saturated by both drugs; indeed, values of the dissociation equilibrium constant for ibuprofen and warfarin binding to HSA-heme-Fe(III) are ≤1×10^−3^ M (at pH 6.5 to 7.5 and 20.0 to 25.0°C) [11], [14], [25]–[27].

All the other chemicals were obtained from Sigma-Aldrich and Merck AG (Darmstadt, Germany). All products were of analytical or reagent grade and were used without further purification.

## Methods

### 3.1. Fe-K edge X-ray absorption spectroscopy of HSA-heme-Fe(III)

Fe-K edge (E_Fe_ = 7.112 keV) X-ray absorption spectra of HSA-heme-Fe(III) in the absence and presence of ibuprofen or warfarin were collected at the BM23 XAFS beamline (Exp. Number: MX1275) at the European Synchrotron Radiation Facility (ESRF, Grenoble, France) in fluorescence geometry. The beam line optics is equipped with a double crystal, fixed exit, Si[111] monochromator. A couple of Si mirrors ensures efficient harmonic rejection and vertical focusing X-ray beam, the size of X-ray spot on the sample was 0.5 mm (vertical)×4 mm (horizontal). The beam energy was calibrated and monitored during the measurements determining the absorption spectra of a Fe reference metal foil placed after the sample.

Solutions of HSA-heme-Fe(III) in the absence and presence of ibuprofen or warfarin were enclosed in a plexiglass cell with Kapton windows: 7 mm (vertical)×12 mm (horizontal). HSA-heme-Fe(III) solutions were cooled around 20 K to preserve the samples from Fe(III) photo-reduction [28], and to reduce the thermal contribution to the structural disorder [24], [29]. The incident intensity (*I*_o_) was measured using an Ar filled ionization chamber. The Fe-K_α_ fluorescence yield (E(*k*_α_)∼6.40 keV) was measured using a 13-elements ultra-pure Ge multi-detector, the total fluoresce signal (*I*_f_) was calculated as a sum over all the detector signals. The Fe-K edge absorption signal was calculated as follows (Eq. 1): 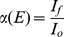
(1)


In order to check the integrity of sample and monitor the stability of the Fe valence state upon X-ray irradiation before the experiment run, several XANES spectra were collected on a HSA-heme-Fe(III) sample as a function of time. Remarkably, sizable photo-reduction is appreciable only more than five hours of sample exposure (Fig. S1 in File S1). Several spectra of each sample were measured, fixing to 1.5 hours the collection time for each spectrum *I*_i_(E). All HSA-heme-Fe(III) samples were shifted vertically by 0.8 mm after each spectrum in order to collect data from unexposed portions of the sample. Up to 14 spectra (*N*) were collected for each HSA-heme-Fe(III) sample and averaged up (
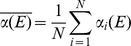
) to obtain reliable, high quality XAS data. The average statistical noise of the averaged Fe-K edge XAS spectra has been calculated according to literature [30] resulting in the 0.9×10^−3^ to 1.2×10^−3^ range.

The averaged spectra 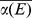 were treated using standard procedures for background subtraction, as well as normalization and extraction of the extended X-ray absorption fine structure (EXAFS) signal 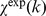 [24]. Briefly, the pre-edge absorption has been calculated as a regression line, and the post-edge background has been modelled with first derivative continue polynomial splines through the data [30]. The origin of the photoelectron energy scale (*E*_o_) has been chosen at the first inflection point of the edge (first derivative maximum) and the energy shift (ΔE) was refined during data analysis [30]. The photoelectron wavevector has been defined as: 
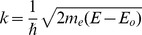
, where *m*_e_ is the electron mass.

The EXAFS data analysis has been carried out using the FitEXA program [30] which exploits the versatility of the MINUIT subroutines [31] for a non-linear least-square fitting procedure and best fit statistical analysis. Least-square refinement procedures have been performed in the reciprocal (*k*) space fitting the raw *k*-weighted spectra 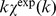 according to the theoretical XAFS equation (Eq. 2) [29]:
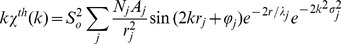
(2)


Equation 2 reproduces the EXAFS spectrum as a sum of partial contributions calculated assuming a Gaussian model for the *j*-th neighbor shell that is defined via *N*_j_, *r*_j_, and 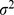 structural parameters, corresponding to the multiplicity (coordination number), the interatomic distance, and the variance (mean square relative displacement, MSRD) of the *j*-th neighbor shell, respectively. Moreover, 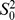 is an empirical parameter taking into account for many body losses; 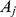, 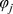, and 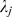 are the photoelectron amplitude, the phase shift, and the mean free path functions, respectively. These functions have been calculated *ab initio* using the FEFF program [32] and the X-ray crystal structures of HSA-heme-Fe(III); notice that both structures deposited within the Protein Data Bank (PDB codes: 1O9X and 1N5U) give definitively similar HSA-heme-Fe(III) environment and can be used indifferently [21], [22]. Complex Hedin-Lundqvist exchange-correlation potentials [33] were used fixing the constant imaginary part to 0.6 eV, no further correction to the theoretical mean free path terms have been added in the analysis. The quality of the fit has been evaluated by the reduced 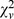 function and the squared residual function: 
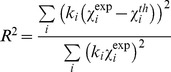
, the sum running over the experimental data points [30]. Values of the standard error of the refined parameters have been calculated using the MINOS option in MINUIT, which takes into account for the correlation effects among the parameters [31]. In order to check the consistency of the data and the refinement procedure, the EXAFS data analysis proceeded firstly fitting the HSA-heme-Fe(III) EXAFS spectrum starting from the known crystallographic structure [21], [22]. This allowed defining the empirical parameters 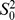 = 0.85 and the edge energy shift ΔE = 1.35 eV which are kept fixed in the analysis of drug-bound HSA-heme-Fe(III) samples.

### 3.2. Steered molecular dynamic simulations of HSA-heme-Fe(III)

Domain I of HSA-heme-Fe(III) (DI; residues 1–195, from PDB code 1O9X) [22], containing the FA1 site, has been used for SMDS. The missing side chains of residues Lys12, Lys41, Lys73, Glu82, Tyr84, Glu86, Glu100, Asp108, Asn109, Lys136, Arg144, Lys159, Gln170, Lys181, and Arg186 have been added using the PRIME module of the MAESTRO suite [34].

The resulting structure of DI of HSA-heme-Fe(III) has been minimized using the Macromodel module of the MAESTRO suite [35]. At this stage, Tyr161 was deprotonated with a negative charge on the oxygen phenoxy atom and a distance constrain was imposed between the oxygen phenoxy atom of Tyr161 and the heme-Fe(III) atom of DI of HSA-heme-Fe(III). The distance imposed was of 2.8 Å with a force of 100 kJ mol^−1^ Å^−1^. The structure has been minimized with the Polak-Ribière Conjugate Gradient method until the gradient reached the threshold of 0.05 kJ mol^−1^ Å^−1^. The refined structure of D1 of HSA-heme-Fe(III) has been used as an input for all atom simulations using the GROMACS package, version 4.5.5 [36], with the GROMOS 53A6 force field [37] and the SPC water model [38]. These parameters were previously used and validated in the simulations of heme-containing systems as reported in [39]–[42]. The parameters for the deprotonated Tyr161 residue coordinating the heme-Fe(III) atom have been determined by the ATB program [43]. The reliability of the parameters for the tyrosinate ion was checked by control MD runs, followed by analysis of the stability of the structural properties of the system with the Fe(III) tyrosinate coordination. The refined structure of D1 of HSA-heme-Fe(III) has been placed in a cubic box big enough to have 1.0 nm space in each direction from the protein. The D1 of HSA-heme-Fe(III) has been solvated with 16720 water molecules. The protonation states of the ionizable amino acids were chosen to be compatible with neutral pH, resulting in a total negative charge (−11) of D1 of HSA-heme-Fe(III). Electroneutrality has been guaranteed by the addition of 11 Na^+^ cations. The coordination distance between the charged oxygen phenoxy atom of Tyr161 and the heme-Fe(III) atom [22] has been kept around the experimental value in MD using a harmonic constrain based on the ensemble averaging during the MD simulation using the distance restrain option of GROMACS package [36]. The force constant was set to 800 kJ mol^−1^ Å^−2^ with an equilibrium distance of 2.7 Å.

To favor the reorientation of the α-helix spanning Glu131-Arg145 and to pull the imidazole group of His146 into the coordination sphere of the heme-Fe(III) atom, a steered MD approach was used through the STEER MD module of the PLUMED plug-in [44]. The helical conformation of the Glu131-Arg145 stretch was preserved through a restrain of 800 kJ mol^−1^ Å^−2^. The target distance between the His146 Nε atom and the heme-Fe(III) atom has been set to 3 Å. To pull the His146 side chain into the coordination sphere of the heme-Fe(III) atom, a force of 80 kJ mol^−1^ Å^−2^ has been used. This force has been applied with a velocity of 0.01 nm ps^−1^ which results in a slow perturbation allowing the protein to adapt to the new conditions. Once the target final structure was reached, without major distortions in the secondary structures of D1, a bond between the heme-Fe(III) atom and the His146 Nε atom was imposed using the GROMOS 53A6 parameters. The resulting structure with the His-Fe bond was further simulated for 15 ns in explicit water as described above, in the absence of any additional restraints.

In all simulations, the electrostatic term has been described by using the particle mesh Ewald algorithm [45]. The LINCS algorithm [46] has been used to constrain all bond lengths. For the water molecules, the SETTLE algorithm [47] has been used. A dielectric permittivity of 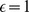 and a time step of 2 fs have been used. The initial velocity of all atoms has been obtained from a Maxwellian distribution at the desired initial temperature of 300 K. The density of the system has been adjusted performing the first equilibration runs at constant number of particles, pressure, and temperature (NPT) conditions by weak coupling to a bath of constant pressure (*P*_0_ = 1 bar, and coupling time τ_p_ = 0.5 ps). In all simulations, the temperature has been maintained close to the intended values by weak coupling to an external temperature bath [38] with a coupling constant of 0.1 ps. The protein and the rest of the system have been coupled separately to the temperature bath.

The .pdb file of the final proposed model, minimized after MD together with the force field parameters and the topology for the protein and the prosthetic group are provided in File S2.

## Results

**Figure 2:**
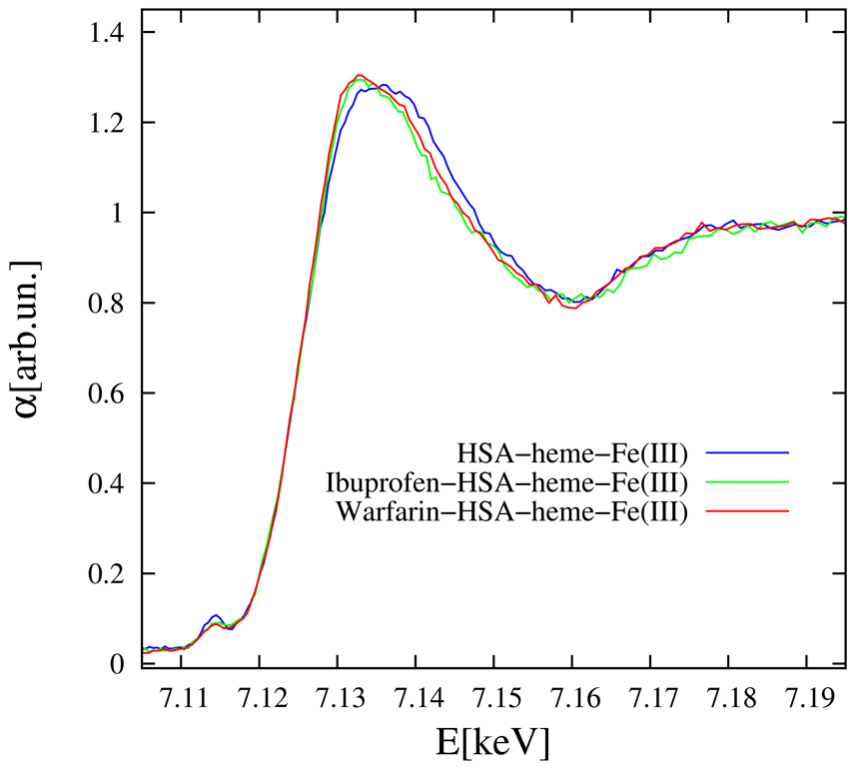
XANES data: comparison of Fe-K edge XANES measured on HSA-heme-Fe(III), ibuprofen-HSA-heme-Fe(III), and warfarin-HSA-heme-Fe(III). The effect of drugs is mainly evident at the main XANES peak (arrow) while the edge position is largely unchanged, signaling the same Fe electronic state in HSA and drug added samples. All XAS data were collected at low temperature (around 20 K) and pH 7.0 (1.0×10^−1^ M phosphate buffer).

Figure 2 shows the normalized spectra in the XANES region of HSA-heme-Fe(III) in the absence and presence of ibuprofen or warfarin. Differences between HSA-heme-Fe(III), ibuprofen-HSA-heme-Fe(III), and warfarin-HSA-heme-Fe(III) spectra are particularly evident near to the XANES main peak, around 7.135 keV. Remarkably, the edge position is weakly affected by addition of ibuprofen and warfarin binding to HSA-heme-Fe(III), demonstrating that drugs do not affect the heme-Fe(III) atom electronic state, but mainly modify the atomic structure around it. Since it is difficult to obtain a quantitative structural information from XANES analysis due to the number of electronic and structural parameters involved and the long computation time required [32], [48], the EXAFS region has been firstly focused, allowing a simpler structural interpretation of data in terms of coordination shells [24]. Secondly, the experimental XANES spectra have been compared to *ab initio* models calculated using the HSA-heme-Fe(III) local structure derived combining EXAFS and SMDS results.

### 4.1. EXAFS data analysis

**Figure 3:**
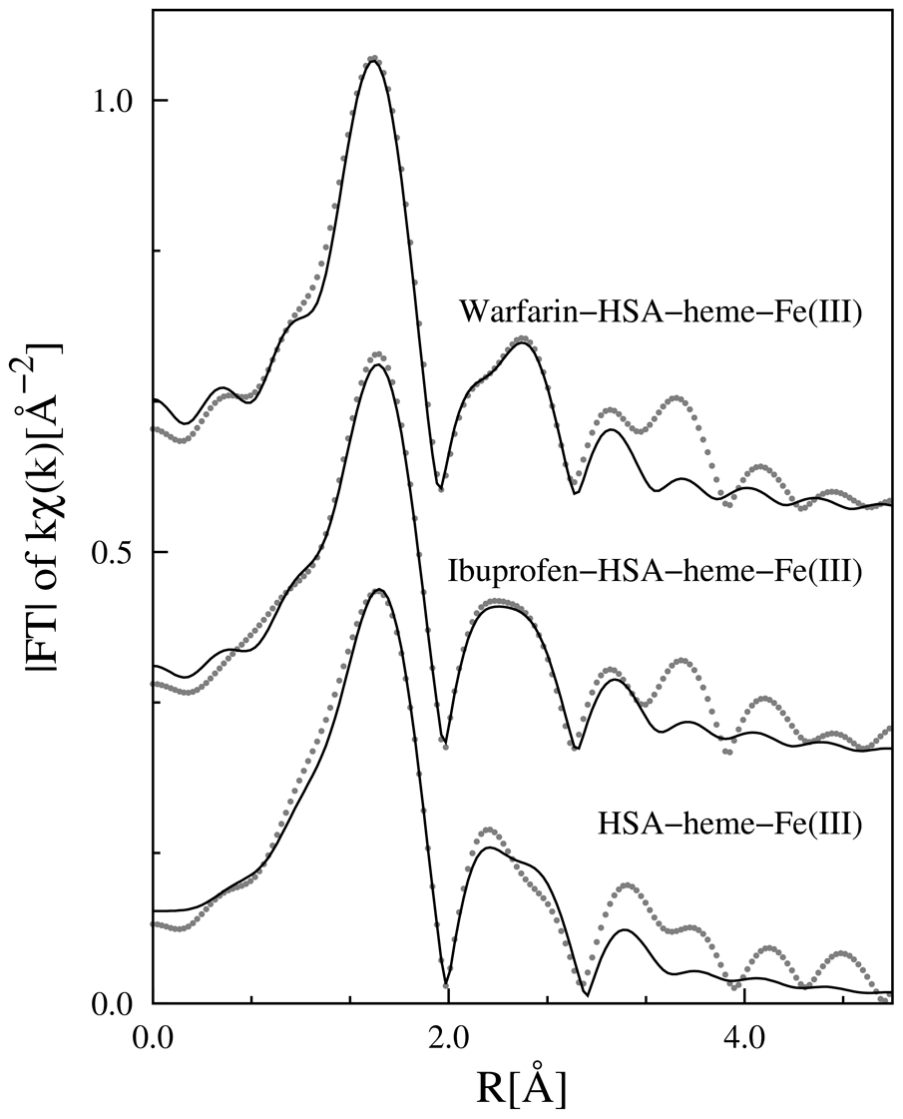
Moduli of the Fourier transform of experimental (dots) and best fit (full line) k-weighted Fe-K edge EXAFS spectra of all the investigated samples (vertically shifted for sake of clarity). The FT are prone to simple qualitative interpretation of the local structure around the absorber as the peaks denote a coordination shell. However, the phase shift function (Eq. 1) reduces the peak position by roughly 0.5 Å with respect to the real value.

The Fourier transform (FT) moduli of k-weighted EXAFS signals (Fig. 3) provide a first qualitative characterization of the local structure around the heme-Fe(III) atom as the FT peaks denote interatomic coordination distances. Notably, the FT peak positions appear compressed by roughly 0.5 Å with respect to the true interatomic distances due to the phase shift term in the EXAFS formula (Eq. 1) [29]. The FTs show an intense first peak around 1.5 Å originating mainly from the nearest neighbor heme-Fe(III) coordination shells. Moreover, evident peaks are observed up to 4 Å and structural modifications occurring around the heme-Fe(III) atom upon ibuprofen and warfarin binding can be hypothesized by examining the differences observed among the drug-free and the drug-bound HSA-heme-Fe(III) (Fig. 2).

The five-coordination state of the heme-Fe(III) atom observed by X-ray crystallography [21], [22] corresponds to what reported in solution by ^17^O-NMR spectroscopy [49]. Therefore, the crystallographic structure of the HSA-heme-Fe(III) [21], [22] has been used as the starting point for the quantitative EXAFS data analysis. In the HSA-heme-Fe(III) crystal structure [21], [22], the heme-Fe(III) atom coordination is largely distorted with four heme nitrogen atoms (Fe-N_I_) of the macrocycle at *R*(Fe-N_I_)∼2 Å (shell I) and one additional bond with the oxygen phenoxy atom of the Tyr161 residue (Fe-O_T_) at *R*(Fe-O_T_)∼2.7 Å (shell II). These shells correspond to the main FT peak around 1.5 Å (Fig. 3). Due to the large difference between *R*(Fe-N_I_) and *R*(Fe-O_T_) distances the five-fold coordination state of the heme-Fe(III) atom has been denoted as 4+1-coordination.

The bonding geometry of the Tyr161 side chain is supposed to be quite flexible, then we hypothesize that the major contribution to the next neighbor FT peaks comes from the heme structure which is relatively rigid. Therefore, the eight carbon atoms (C_2_) form a coordination shell (shell III) at the average distance *R*(Fe-C_2_)∼3.1 Å, and the four carbon atoms (C_3_) give rise to a coordination shell (shell IV) at the average distance *R*(Fe-C_3_)∼3.4 Å (Fig. 1). These two shells must produce the second FT peak observed around 3–3.5 Å (Fig. 3). The third main peak in the FT, observed around 3.8 Å, must be associated to the more distant shell of the C_4_ ions located at *R*(Fe-C_4_)∼4.3 Å. Noticeably, strong multiple scattering (MS) effects are expected to occur in this region, due to the almost collinear Fe(III)-N_I_-C_4_ configurations (bond angle θ_N1_∼170°) [50], [51]

A good fitting of HSA-heme-Fe(III) EXAFS data has been obtained by considering the shells I, II, III, and IV (without the MS shell), keeping the coordination numbers fixed accordingly to the crystallographic structure of the FA1 site, and refining the average shell distances (*R*_j_) and MSRD parameters (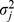). Attempts to include contributions from the C_4_ shell in the analysis of the EXAFS spectra worsened the correlation among the fitting parameters (as also noticed in ref. [51]) and the reliability of the refinement. Therefore, the analysis has been restricted to the first four shells excluding the C_4_ shell, however this does not affect the information about heme-Fe(III) coordination mode.

**Figure 4:**
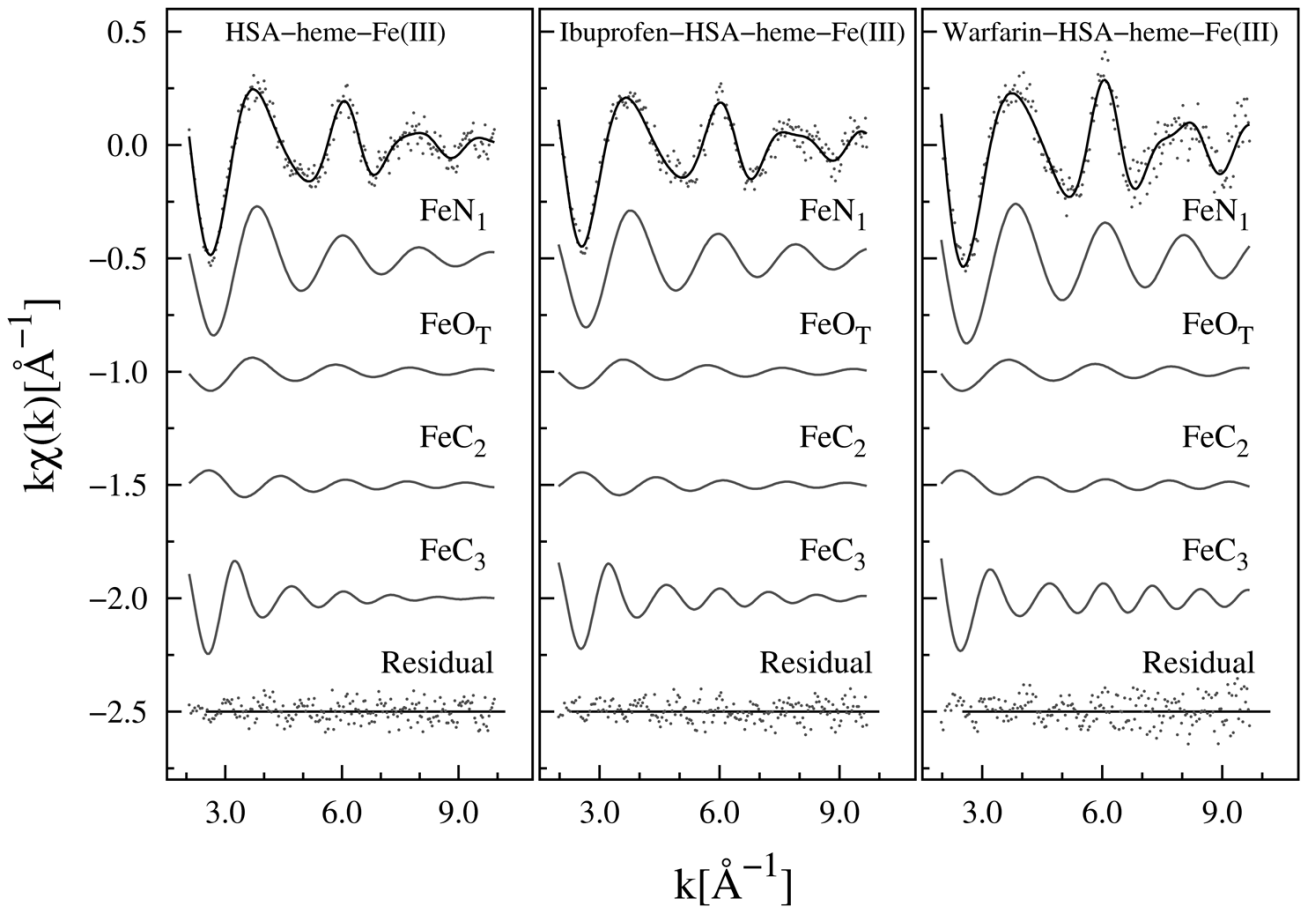
EXAFS data fitting. Experimental data (points) and best fit (full lines) for all the analyzed samples are shown. The partial contributions (shells) used for the refinement of each spectrum are shown (vertically shifted for clarity); the structural parameters are reported in Table 1. The residuals (experimental data minus best fit) are shown, at the bottom for each sample.

Figure 4 shows the k-weighted experimental EXAFS data (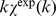) along with the best fit curves (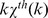). The contributions used for the refinement, the residual values, and the structural parameters are listed in Table 1. The coordination distances of the heme-Fe(III) atom in the HSA-heme-Fe(III) complex are in good agreement with those determined by X-ray crystallography [20], [21], giving confidence on the present analysis. Noticeably, the Fe-O bond results compressed (*R*(Fe-O_T_) = 2.5 Å) with respect to that shown in the crystallographic model (*R*(Fe-O_T_) = 2.78 Å) while the Fe-C_2_ shell distance obtained by EXAFS is slightly (2%) expanded with respect to that reported in the crystallographic model. This discrepancy may reflect the solution and crystal state of HSA-heme-Fe(III). Note that in the multiple shell data fitting procedure, the consistency of the structural parameters of the different shells, and the possibility to constraint structural parameters (*i.e.*, energy shift and coordination numbers) to geometrical models is helpful to strength the reliability of the results, specially dealing with relatively noisy data [50]–[53].

**Table 1:** Local atomic structure of HSA-heme-Fe(III), ibuprofen-HSA-heme-Fe(III), and warfarin-HSA-heme-Fe(III) around the heme-Fe(III) atom as obtained by EXAFS data analysis, compared with the average coordination distances of models (PDB and SMD).

HSA-heme-Fe(III)							
	PDB Model		EXAFS results			XANES model	
shell	N	R	N	R	σ^2^×10^3^	N	R
		[Å]		[Å]	[Å^2^]		[Å]
I Fe-N	4	2.06	4 a	2.07(1)	4.1(2)	4	2.07
II Fe-O	1	2.78	1 a	2.48(2)	1.1(2)	1	2.54
III Fe-C_1_	8	3.07	8 a	3.09(2)	13(3)	8	3.08
IV Fe-C_2_	4	3.43	4 a	3.50(3)	5(1)	4	3.55
Fe-C_4_	8	4.28	-	-	-	8	4.23
			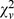	1.36			
			R^2^	0.108			

The standard uncertainty on the parameters refined for the EXAFS data analysis are reported in parenthesis (last digit variation). The 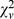 and R^2^ parameters (EXAFS analysis) are reported (see text). The last right columns contain the number of neighbors and average distance of the structure used for XANES models. The C_4_ coordination shell is included in the XANES simulations.

a Coordination numbers were fixed to the crystallographic values. Noticeably, refining N_I_ and N_II_ in HSA-heme-Fe(III) data, they changed less than 10% well within the estimated uncertainty. Therefore, we can safely assume correct the five-coordination of the heme-Fe(III) atom in the HSA-heme-Fe(III)-complex in solution fixing N_I_ = 4 and N_II_ = 1.

Optical spectroscopy investigations [11], [13], [14], [19], [20] and the SMDS model (see below) suggest that ibuprofen and warfarin binding would lead to the formation of an additional ligand of the heme-Fe(III) atom. Changes observed looking at the XAFS data (in particular XANES and FTs) highlight drug effects on the local structure of HSA-heme-Fe(III). Note that in standard EXAFS data analysis, the additional neighbor suggested by optical spectroscopy and the SMDS model can be hindered owing the correlation among the fitting parameters. Firstly, the ibuprofen and warfarin bound HSA-heme-Fe(III) EXAFS data have been analyzed fixing N_I_ = 4 and N_II_ = 1 and leaving 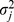 free to vary with a lower limit at 1/4 of the values found in pure HSA-heme-Fe(III). The best fit shows that 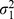 (first shell) systematically reaches the lower limit (imposed to 1×10^−3^ Å^2^) still providing an unsatisfactory fit (being 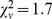 and R^2^ around 0.15; Fig. S2 in File S1). Secondly, an additional Fe-N shell (Fe-N_b_) has been added in order to verify the possibility of the additional heme-Fe(III) neighbor. Despite the uncertainty on N(Fe-N_b_) is relatively large (about 50%), this contribution improves the best fit quality giving confidence on this finding. Noticeably, the heme-Fe(III) next neighbor distances change weakly upon drug addition but their disorder factors decrease systematically.

### 4.2. SMDS model

**Figure 5:**
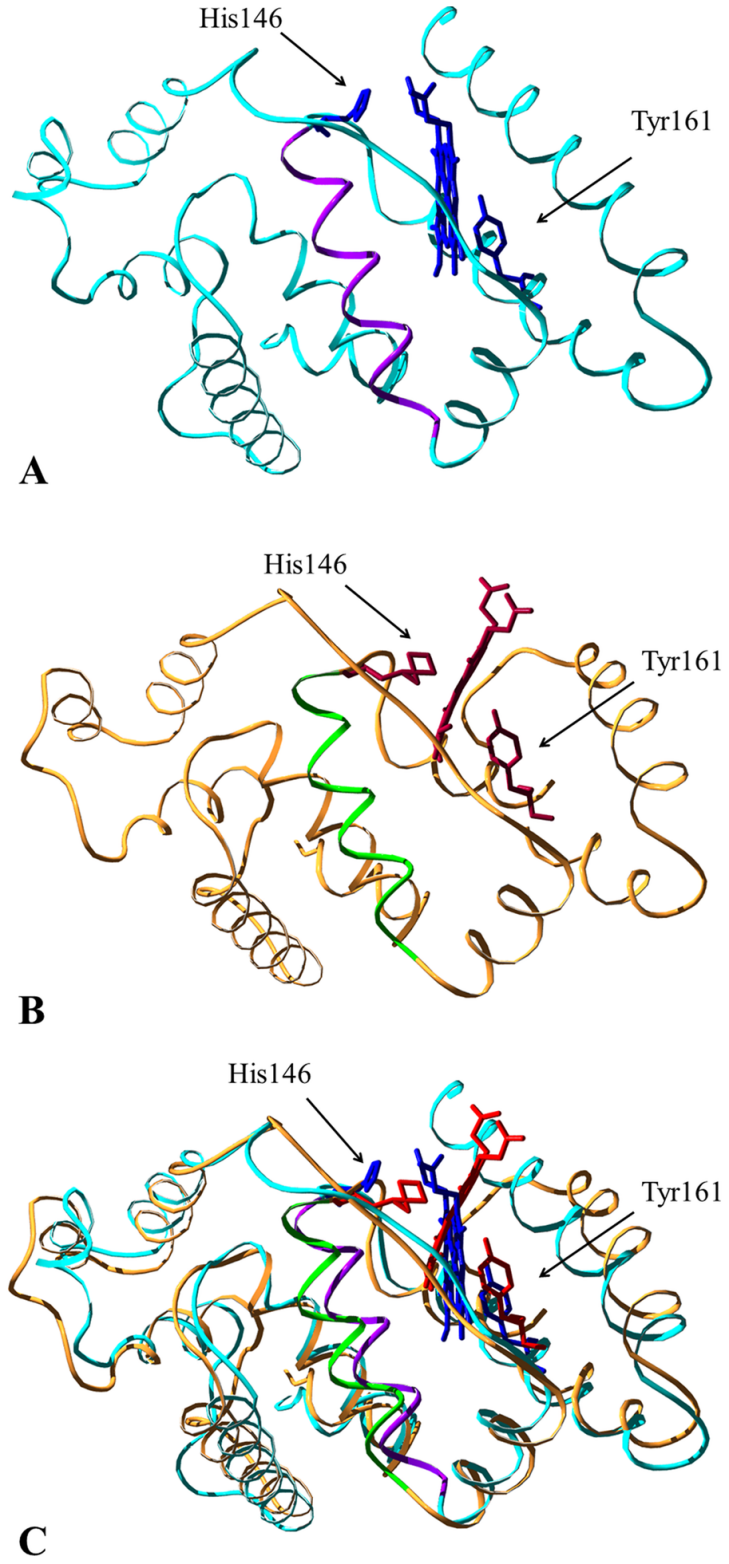
Conformational transition of HSA-heme-Fe(III) upon ligand binding to the FA2 site. Panel A. Three-dimensional representation of the starting crystal structure (cyan, PDB entry 1O9X [22]) of HSA-heme-Fe(III). Heme-Fe(III) and the His146 and Tyr161 residues are highlighted in blue. The Glu131-Arg145 α-helix is represented in magenta. Panel B. Three-dimensional representation of the final model (orange) of HSA-heme-Fe(III) obtained via SMDS. Heme-Fe(III) and the His146 and Tyr161 residues are highlighted in red. The Glu131-Arg145 α-helix is represented in green. Panel C. Superposition of the starting crystal structure and of the final model of HSA-heme-Fe(III). The picture has been drawn using the UCSF Chimera package [67], [68].

In order to build a HSA-heme-Fe(III) model able to accommodate a six-coordinated heme-Fe(III) atom and to understand a possible origin for the sixth heme-Fe(III) bond, SMDS have been used. The target structure involves the His146 coordinated to the heme-Fe(III) atom at the sixth coordination position (Fig. 5), providing an additional Fe-N interatomic distance of about 1.98 Å, definitively in agreement with the increased number of nearest neighbors found by EXAFS analysis. As His146 approaches the heme-Fe(III) atom, a reorganization of the loop spanning residues 146–150 can be observed. This is accompanied by the translation of the Glu131-Arg145 α-helix, which has to move in a rigid-body like fashion to accommodate the new position of His146. Interestingly, SMDS results show that it is possible to obtain the structural organization consistent with the six-coordination state of the Fe(III) atom. In order to check the structural viability of this new conformation and to provide a qualitative picture of the initial events correlated to the allosteric conformational change(s), a direct Fe-His bond has been imposed on the structure obtained at the end of SMDS. The resulting model has been next simulated using classical MD simulations without any restraints following the scheme detailed in Materials and Methods. Evaluation of the time-dependent root mean square positional deviation (RMSD) of the backbone atoms from the initial model (obtained with SMD) as a function of the simulation time shows that after some rearrangements related to the release of strain generated in the Steering stage, the protein stabilizes in a stable structural ensemble after about 4 ns (Fig. S3– in File S1). Analysis of the time dependent evolution of the secondary structure content, according to the DSSP algorithm, also confirms the overall stability of the obtained structure. The proposed model is shown in Figure 5.

Table 1 shows the main parameters characterizing the heme-Fe(III) atom local structure as obtained from SMDS. The SMDS parameters agree with those obtained from EXAFS data analysis, proving the consistency of the structural model. The atomic coordinates of HSA accommodating a six-coordinated heme-Fe(III) atom are reported in File S2.

### 4.3. XANES modelling

**Figure 6:**
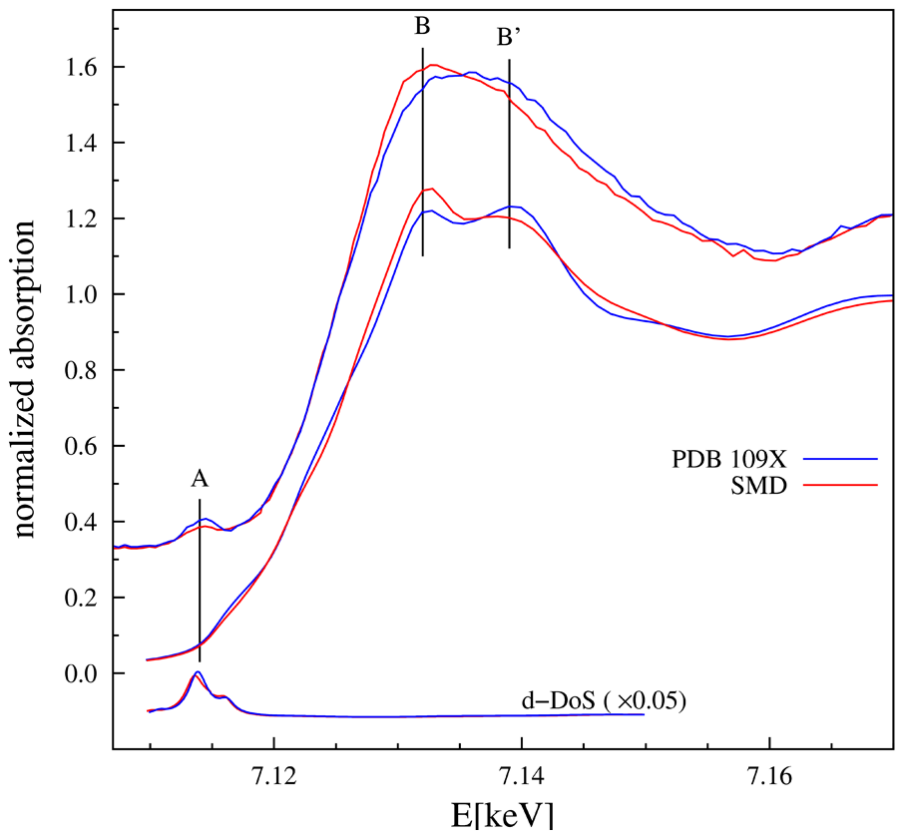
Top curves represent the experimental XANES spectra of HSA-heme-Fe(III) (blue) and warfarin-HSA-heme-Fe(III) (red). Middle curves are the XANES spectra calculated *ab initio* using the FEFF code [32] for atomic cluster made combining SMDS and EXAFS results. The bottom curves represent the density of *d* states (*d*-DoS). The calculated XANES spectra reproduce the main experimental features, in particular the inversion of B and B′ peaks upon drug binding is reproduced. The pre-edge peak A corresponds to the *d*-DoS.

In order to have a further confirmation *ab initio* full multiple scattering (FMS) XANES spectra were calculated using the FEFF 8.1 program in muffin-tin approximation, using self consistent Hedin-Lundqvist exchange potentials with 0.6 eV constant imaginary term. Atomic clusters of 4.5 Å radius around the heme-Fe(III) atom were used for FMS. Noticeably, the calculated XANES spectra reproduce the main features of the experimental spectra and the modifications observed upon drug addition, in particular the shape of the “white line” of the theoretical XANES spectra change accordingly to the experimental spectra (Fig. 6). We must notice here that we used the structural information derived from EXAFS analysis (Table 1) to slightly modify the local structure around heme-Fe(III) atoms obtained from the SMDS simulations. In particular, the Tyr161 residue has been moved close to the heme-Fe(III) atom to make the Fe-O distance of *ca.* 2.5 Å, and the Fe-N_I_ shell of the SMDS model has been expanded. The average shell distances used for the XANES model are given in Table 1.

**Figure 7:**
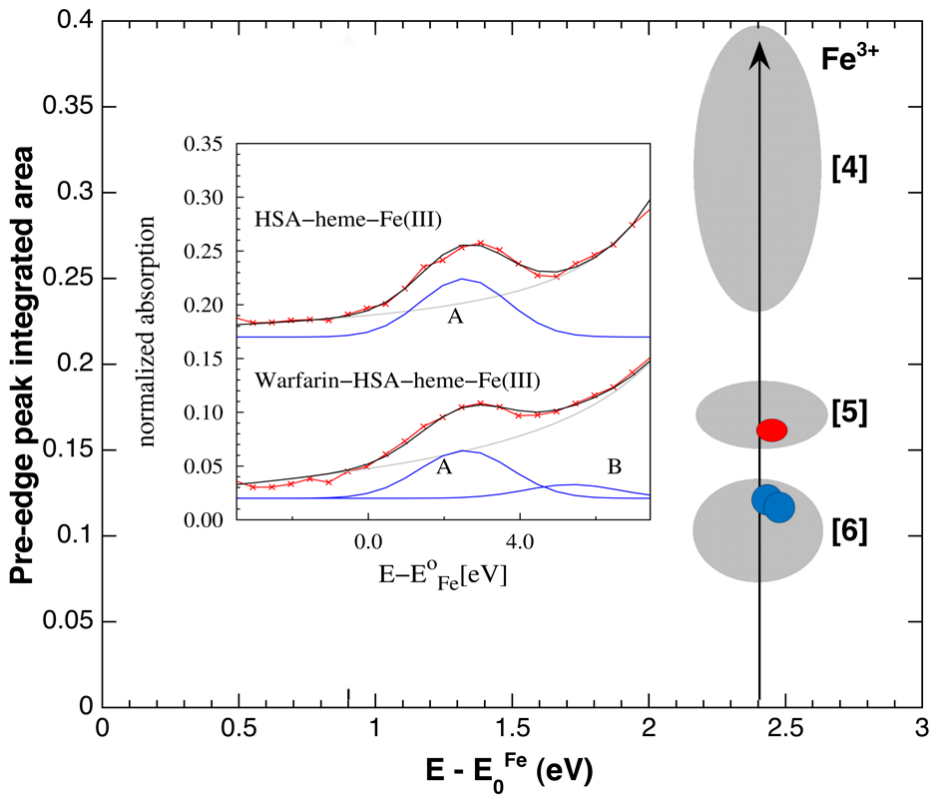
Semi-quantitative analysis of Fe(III) pre-edge peaks and comparison with reference compounds. In the inset, the experimental Fe(III) pre-edge peak (red symbols) of HSA-heme-Fe(III) and warfarin-HSA-heme-Fe(III) are modelled combining an arctangent function (gray line), simulating the onset of the continuous electron states, and Gaussian peaks (blue lines), representing the transitions to localized electronic states. The grey ellipses enclose the values of Fe(III) pre-edge peak integrated areas versus their centroid positions, measured on tetra-, penta-, and hexa- coordinated Fe(III) reference compounds (labelled [4], [5], and [6], respectively; [56]). The pre-edge peak parameters of HSA-heme-Fe(III) (red circle) fall in the region of penta-coordinated Fe(III), while ibuprofen-HSA-heme-Fe(III) and warfarin-HSA-heme-Fe(III) pre-edge peaks parameters (blue circles) are in the region corresponding to hexa-coordinated Fe(III) compounds. The pre-edge fitting of drug-bound HSA-heme-Fe(III) samples requires an additional peak (labelled B) in the region about 2 eV above the main one (labelled A). Analogous components have been reported in ref. [54]. The size of red and blue symbols takes into account for best fit incertitude.

As shown in Figure 6, the calculated XANES spectra do not reproduce the experimental data in the pre-edge region. This is not surprising because in this region the spectral features crucially depend on the finest details of interatomic potentials and crystal field effects, so that the main approximation for XANES calculations may result inadequate, specially dealing with strongly asymmetric and distorted structures [54], [55]. Here, the pre-edge features of the heme-Fe(III) atom originate from electronic transitions to localized Fe states close to the Fermi level having *d*-symmetry [54]. Of note, the calculated density of the *d*-states (*d*-DoS) (Fig. 6) corresponds to the experimental pre-edge peak. In the case of 6-coordinated Fe(III) atom in the regular octahedral symmetry, the pre-edge is weak as originating from quadrupole *s*-*d* transitions [54], [55]. However, local distortions promote some *p*-*d* hybridization enhancing the pre-edge peak due to dipole allowed transition to hybrid *p*-*d* states. Although the modeling of *p*-*d* hybridization requires deeper theory [54] which is beyond the aim of this study, relevant information about heme-Fe(III) atom coordination chemistry can be obtained comparing the integrated area and position of Fe(III) pre-edge peaks with those of reference compounds [56]. As shown in Figure 7, HSA-heme-Fe(III) data fall in the region of penta-coordinated Fe(III) complexes, while ibuprofen-HSA-heme-Fe(III) and warfarin-HSA-heme-Fe(III) data are consistent with those of hexa-coordinated Fe(III) compounds. Accounting for the drug concentration used (1.0×10^−2^ M) and the dissociation equilibrium constants for ibuprofen and warfarin binding to the allosterically-coupled FA2 site of HSA-heme-Fe(III) (*ca.* 1.0×10^−3^ M; see below), ibuprofen- and warfarin-containing samples may still comprise up to 10% of penta-coordinated drug-free HSA-heme-Fe(III).

## Discussion

Binding of ibuprofen and warfarin, in the millimolar concentration range, to HSA(-heme-Fe(III)) induced a dramatic conformational rearrangement of the heme-Fe(III) coordination sphere. As thoroughly reported, ibuprofen and warfarin modulate allosterically and competitively HSA(-heme-Fe(III)) spectroscopic and reactivity properties by binding to multiple sites [1], [2], [11]–[13], [16]–[18]. In particular, these prototypical drugs: (*i*) inhibit peroxynitrite isomerization by HSA-heme-Fe(III), facilitating the peroxynitrite-mediated nitration of free L-tyrosine [11], [19]; (*ii*) impair NO binding to HSA-heme-Fe(II), lowering the value of second-order combination rate constant and increasing the value of first-order dissociation rate constant [1], [12], [57]; and (*iii*) affect UV-visible, resonance Raman, and electron paramagnetic resonance spectroscopic properties of HSA-heme-Fe(III), HSA-heme-Fe(II)-CO, and HSA-heme-Fe(II)-NO [2], [19], [25], [58]. These effects have been suggested to be a consequence of the six-coordination of the heme-Fe atom of HSA-heme-Fe(III), HSA-heme-Fe(II), HSA-heme-Fe(II)-NO, and HSA-heme-Fe(II)-CO, following ligand (*e.g.*, drug) binding to FA2 [11]–[13], [19].

Crystallographic and solution studies indicate distinct binding modes and dissociation equilibrium constants for both ibuprofen and warfarin. Ibuprofen binds to two main HSA region as observed by X-ray diffraction, approximately identified as FA3–FA4 and FA6; however, values of the dissociation equilibrium constants for ibuprofen binding to HSA-heme-Fe are: *K*_1_ = 3.1×10^−7^ M, *K*_2_ = 1.7×10^−4^ M, and *K*_3_ = 2.2×10^−3^ M [10], [59]. In a similar way, warfarin binds to its primary site, corresponding to FA7; however, values of the dissociation equilibrium constants for warfarin binding to HSA-heme-Fe are: *K*_1_ = 5.3×10^−5^ M and *K*_2_ = 5×10^−4^ M [10], [14]. Therefore, the comparison between crystallographic and thermodynamics investigations suggests the occurrence of additional ibuprofen and warfarin binding sites in the HSA-heme-Fe complex with dissociation equilibrium constants in the millimolar range. Since truncated-HSA-heme-Fe(III) (lacking domain III, encompassing the FA3–FA4 cleft and the FA5 site) displays the same allosteric coupling between FA1, FA2, and FA7 as observed in native-HSA-heme-Fe(III) [60], domain III does not regulate the allosteric properties of HSA(-heme-Fe(III)), thus excluding the modulatory role of the primary ibuprofen binding cleft FA3-FA4 and of the FA5 site. Excluding the FA1 site (that binds the heme-Fe), the FA3, FA4, and FA5 sites (which are located in domain III), the FA6 site (which is the secondary, medium-affinity ibuprofen binding site) and the FA7 site (representing the high affinity cleft of warfarin), the additional low-affinity ibuprofen/warfarin binding site can only share the FA2 binding environment. Remarkably, the FA2 site is the binding pocket allosterically coupled to the FA1 cleft [61]. Indeed, FA2 is the only ligand binding site that contacts two HSA domains (namely domains I and II) and is affected in its topology upon FA binding [22].

Although the three-dimensional structures of ibuprofen- and warfarin-HSA-heme-Fe complexes are not available [2], present results are supported by SMDS, demonstrating that the His146 residue is able to orientate toward the heme-Fe(III) atom, as previously proposed [19]. We show that the sixth coordination bond of the heme-Fe(III) atom appears to be at about 2.15 Å, which is consistent with the Fe-N_ε_ bond length reported for heme-Fe-imidazole and -histidine coordination in heme-model compounds and heme-proteins [59]–[66]. Moreover, drug binding reduces the structural disorder in the next neighbor shells of the heme-Fe(III) atom (MSRD of shells III and IV; Table 1), indicating a drug-dependent strong steric constraint of the FA1 site (*i.e.*, of the heme-Fe binding cleft).

It is worth noting that the targeted MD simulations are mainly intended to provide a qualitative view of the initial conformational reorganization steps that lead to change the coordination number of the heme-Fe(III) atom. Indeed, the results indicate that the protein can accommodate the reorientation of the Glu131-Arg145 α-helix. This indicates that a viable structural reorganization mechanism bringing His146 into the coordination sphere of Fe(III) is available on the energy landscape of the protein and may be selected and triggered by the binding of the allosteric effectors ibuprofen and warfarin. This observation is further corroborated by the observation that the model obtained with SMDS is stable at the level of the tertiary and secondary structures when a bond between the His146 residue and the heme-Fe(III) atom is imposed to mimic the six (5+1) coordination observed experimentally. Remarkably, a tilt of the *C*-terminal α-helix (Ala175-Leu185) of the MD simulated model suggests that domains I and II should move apart in a more extended way when compared to the structure of HSA where the FA2 site is occupied by FAs [18]. As the MD simulation was limited to domain I, an intrinsic disorder was observed in residues Arg186-Lys195 that prevented a fine reconstruction of the whole HSA model.

As a caveat, it must be stressed that the results of simulations are qualitative, since, by using classical MD simulation, the observation of the formation/breaking of bonds with electronic reorganization cannot be observed. Importantly, the final Fe-His distance is much shorter than at the start of the simulation (8.7 Å), and His146 is in the correct orientation to coordinate the Fe(III) atom providing a Fe-N_ε_ distance of about 2.2 Å. It is worthwhile to notice that SMDS models are consistent with the experimental findings derived from Fe-K edge XAFS analysis in the extended (EXAFS) and near edge (XANES) regions, strengthening the obtained results. Moreover, a three-dimensional model of HSA-heme-Fe(III), which can be safely assumed as a reliable structural picture of the conformational changes occurring in the ibuprofen- and warfarin-induced allosteric transition, is provided.

As a whole, the Fe-K edge XAS data demonstrate that ibuprofen and warfarin binding to HSA-heme-Fe(III) induces the 4+1 to 5+1 coordination transition of the heme-Fe(III) atom and brings on a strong steric constraint of the FA1 site (*i.e.*, the heme site). Moreover, SMDS indicate that the His146 residue could likely be the additional coordination ligand of the heme-Fe(III) atom of the drug-bound HSA-heme-Fe(III) complexes. Remarkably, the presence of an additional ligand at the sixth coordination position of the heme-Fe atom inhibits its reactivity [2].

Data here reported highlight the role of drugs in modulating HSA functions. This is relevant for the potential role played by HSA-heme-Fe in detoxification processes, also taking into account that the HSA-heme-Fe plasmatic concentration increases significantly under pathological conditions [2].

## Supporting Information

File S1 **Supporting figures. Figure S1, Effect of the X-ray exposure time on normalized Fe-K edge XANES spectra of HSA-heme-Fe(III).** Fe-K edge XANES spectra were measured at low temperature (20 K) as a function of the X-ray exposure time: (i) fresh sample (blue line), (ii) sample exposed for 5 hours (green line), and (iii) sample exposed for 13 hours (red line). The radiation damage is week after 5 hours exposure, but it is evident after prolonged exposure (red line) resulting in the low energy edge shift (arrow). **Figure S2, Analysis of ibuprofen-HSA-heme-Fe(III) EXAFS spectra.** Fourier transform (modulus and imaginary part) of experimental ibuprofen-HSA-heme-Fe(III) EXAFS spectra (dots) are compared with best fit obtained using 5+1 model (top) and 4+1 model (bottom). The 4+1 model definitively worse the fit, moreover the disorder factor for the first shell is anomalously small at the lower imposed limit:  = 1×10−3 Å2. The grey curves are the imaginary part of the Fourier transform of the residuals (experimental data minus best fit): the 5+1 model satisfactorily reproduces the data above R ca. 3 Å. Taking into account for the phase shift effect our model provides an accurate description of Fe(III) local structure up to about 3.5 Å. **Figure S3, Time-dependent evolution of the RMSD of drug-bound HSA-heme-Fe(III).** Data reflect the protein structural reorganization induced by Targeted MD.
(DOC)

File S2 **MD data.** Atomic coordinates of HSA-heme-Fe(III) accommodating a six-coordinated atom. The .pdb file of the final proposed model, minimized after MD, is reported together with the force field parameters and the topology for the protein and the prosthetic group.
(GZ) Click here for additional data file.
