# Correction: The Five-To-Six-Coordination Transition of Ferric Human Serum Heme-Albumin Is Allosterically-Modulated by Ibuprofen and Warfarin: A Combined XAS and MD Study

**DOI:** 10.1371/journal.pone.0123144

**Published:** 2015-03-31

**Authors:** The PLOS ONE Staff

Fig. 5 is incorrect. Please see the corrected Fig. 5 here.

**Fig 5:**
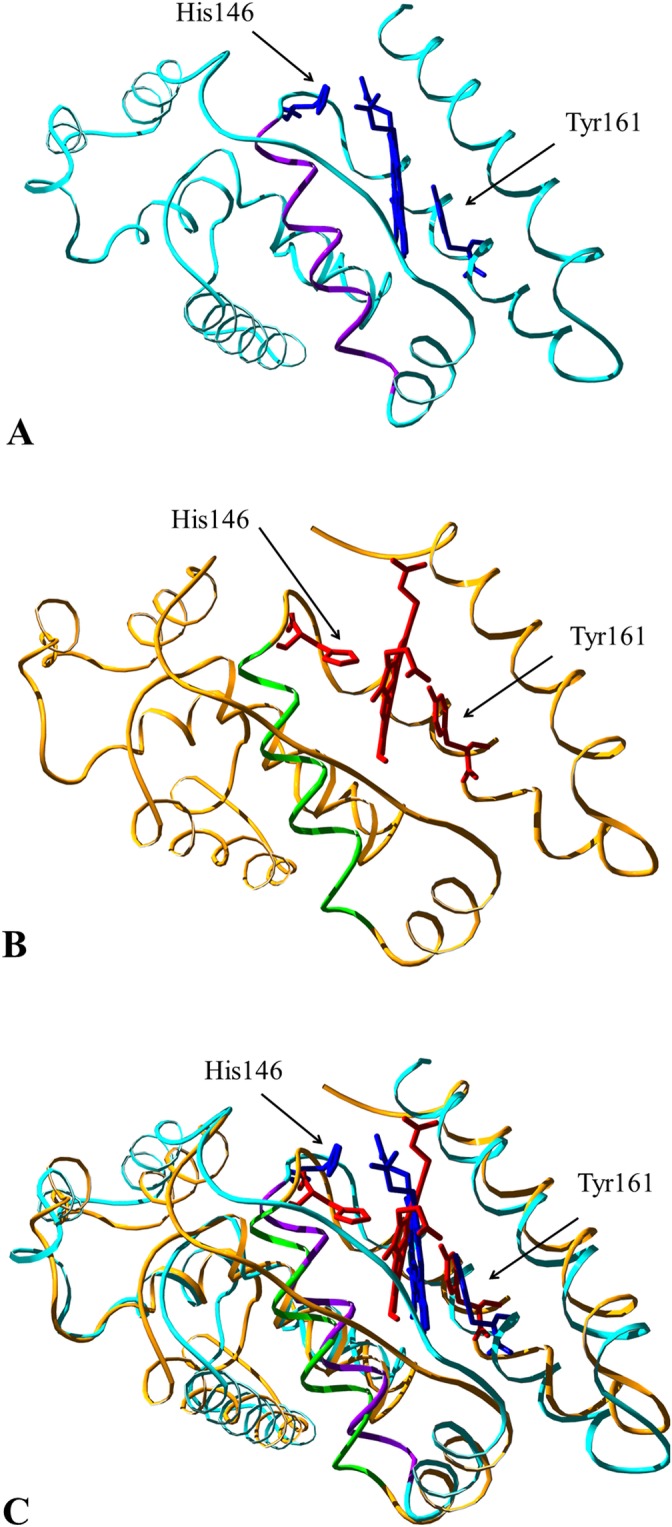
Conformational transition of HSA-heme-Fe(III) upon ligand binding to the FA2 site. Panel A. Three-dimensional representation of the starting crystal structure (cyan, PDB entry 1O9X [22]) of HSA-heme-Fe(III). Heme-Fe(III) and the His146 and Tyr161 residues are highlighted in blue. The Glu131-Arg145 α-helix is represented in magenta. Panel B. Three-dimensional representation of the final model (orange) of HSA-heme-Fe(III) obtained via SMDS. Heme-Fe(III) and the His146 and Tyr161 residues are highlighted in red. The Glu131-Arg145 α-helix is represented in green. Panel C. Superposition of the starting crystal structure and of the final model of HSA-heme-Fe(III). The picture has been drawn using the UCSF Chimera package [67], [68].
