# Supplementary material for: The Five-To-Six-Coordination Transition of Ferric Human Serum Heme-Albumin Is Allosterically-Modulated by Ibuprofen and Warfarin: A Combined XAS and MD Study
Source: PLoS One. 2014 Aug 25;9(8):e104231. doi: 10.1371/journal.pone.0104231 (PMC4143227; doi:10.1371/journal.pone.0104231)
Supplement: File S1 — Supporting figures. Figure S1, Effect of the X-ray exposure time on normalized Fe-K edge XANES spectra of HSA-heme-Fe(III). Fe-K edge XANES spectra were measured at low temperature (20 K) as a function of the X-ray exposure time: (i) fresh sample (blue line), (ii) sample exposed for 5 hours (green line), and (iii) sample exposed for 13 hours (red line). The radiation damage is week after 5 hours exposure, but it is evident after prolonged exposure (red line) resulting in the low energy edge shift (arrow). Figure S2, Analysis of ibuprofen-HSA-heme-Fe(III) EXAFS spectra. Fourier transform (modulus and imaginary part) of experimental ibuprofen-HSA-heme-Fe(III) EXAFS spectra (dots) are compared with best fit obtained using 5+1 model (top) and 4+1 model (bottom). The 4+1 model definitively worse the fit, moreover the disorder factor for the first shell is anomalously small at the lower imposed limit: = 1×10^−3^ Å2. The grey curves are the imaginary part of the Fourier transform of the residuals (experimental data minus best fit): the 5+1 model satisfactorily reproduces the data above R ca. 3 Å. Taking into account for the phase shift effect our model provides an accurate description of Fe(III) local structure up to about 3.5 Å. Figure S3, Time-dependent evolution of the RMSD of drug-bound HSA-heme-Fe(III). Data reflect the protein structural reorganization induced by Targeted MD. (DOC) [file pone.0104231.s001.doc]

**Supporting Information S1**

**The five-to-six-coordination transition of ferric human serum heme-albumin is allosterically-modulated by ibuprofen and warfarin:**

**A combined XAS and MD study**

Carlo Meneghini ^a,#^, Loris Leboffe ^a,b,#^, Monica Bionducci ^a^, Gabriella Fanali ^c^, Massimiliano

Meli ^d^, Giorgio Colombo ^d^, Mauro Fasano ^c^, Paolo Ascenzi ^b,e^,*, and Settimio Mobilio ^a^

^a^ Department of Sciences, Roma Tre University, Roma, Italy

^b^ National Institute of Biostructures and Biosystems, Roma, Italy

^c^ Biomedical Research Division, Department of Theoretical and Applied Sciences, and Center of Neuroscience, University of Insubria, Busto Arsizio (VA), Italy

^d^ Institute for Molecular Recognition Chemistry, National Research Council,

Milano, Italy

^e^ Interdepartmental Laboratory of Electron Microscopy, Roma Tre University, Roma, Italy

^#^ These Authors contributed equally to this work.

* Corresponding authors: Paolo Ascenzi, phone: +39-06-5733 3621; fax: +39-06-5733 6321.


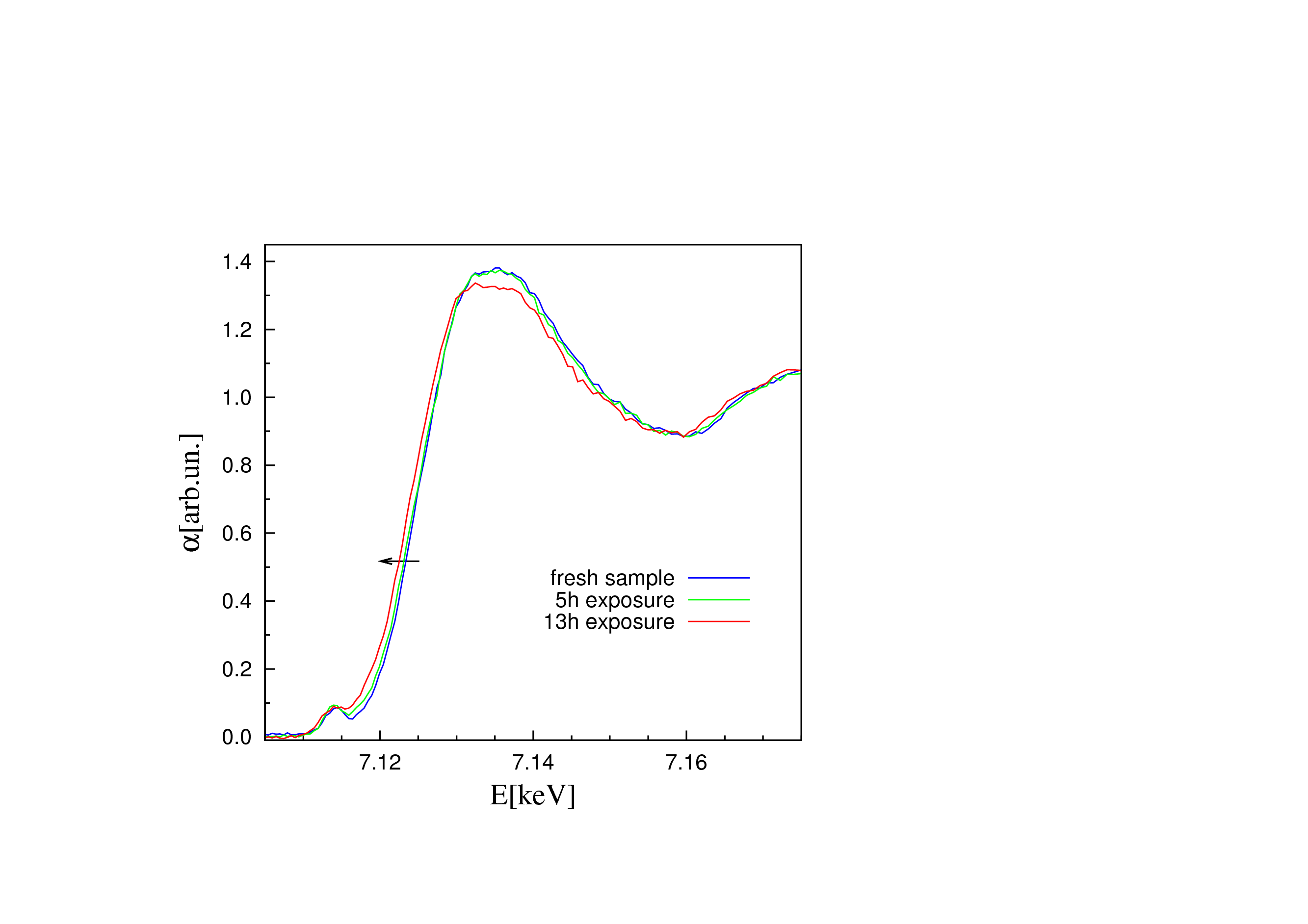


**Fig. S1.** Fe-K edge XANES data: normalized Fe-K edge XANES measured on HSA-heme-Fe(III) at low temperature (20 K) as a function of the X-ray exposure time: (*i*) fresh sample (blue line), (*ii*) sample exposed for 5 hours (green line), and (*iii*) sample exposed for 13 hours (red line). The radiation damage is evident after prolonged exposure (red line) resulting in low energy edge shift (arrow) but it is week after 5 hours exposure.


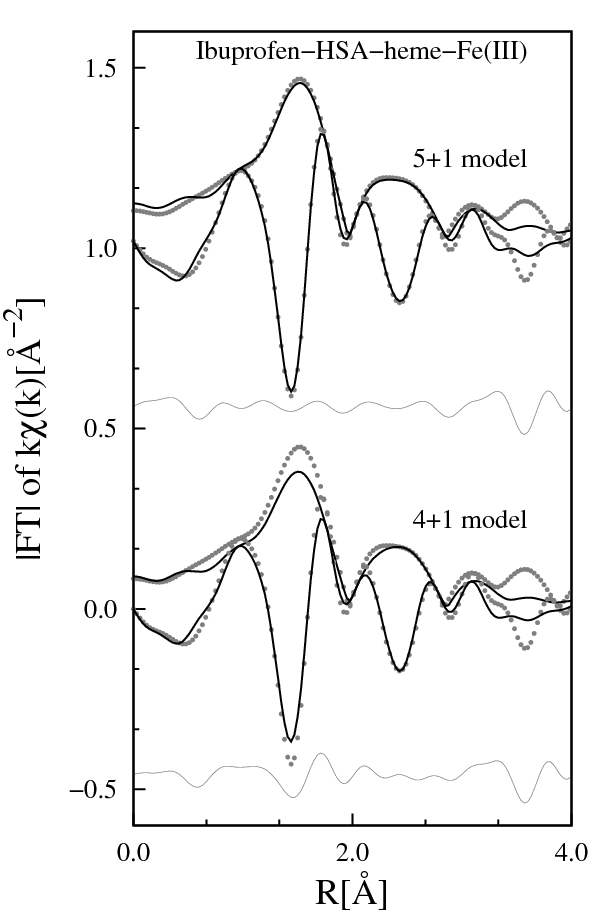


**Fig. S2**. Fourier transform (modulus and imaginary part) of experimental Ibuprofen-HSA-heme-Fe(III) EXAFS spectra (dots) compared with best fit obtained using 5+1 model (top) and 4+1 model (bottom). The 4+1 model definitively worse the fit, moreover the disorder factor for the first shell is anomalously small at the lower imposed limit: $\sigma_{FeN}^{2}$=1×10^-3^ Å^2^. The grey curves are the imaginary part of the Fourier transform of the residuals (experimental data minus best fit): the 5+1 model satisfactorily reproduces the data above R *ca*. 3 Å. Taking into account for the phase shift effect our model provides an accurate description of Fe(III) local structure up to about 3.5 Å.


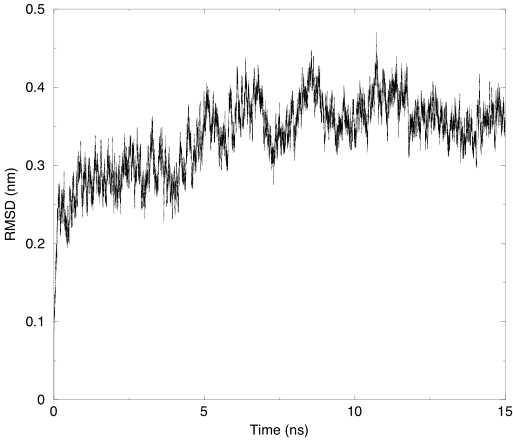


**Fig. S3.** Time dependent evolution of the RMSD from the initial structure of the protein as obtained after the structural reorganization induced by Targeted MD.
